# New *qnr* Gene Cassettes Associated with Superintegron Repeats in *Vibrio cholerae* O1 

**DOI:** 10.3201/eid1407.080132

**Published:** 2008-07

**Authors:** Érica L. Fonseca, Fernanda dos Santos Freitas, Verônica V. Vieira, Ana C.P. Vicente

**Affiliations:** * Instituto Oswaldo Cruz, Rio de Janeiro, Brazil; † Instituto Nacional de Controle de Qualidade em Saúde, Rio de Janeiro

**Keywords:** Quinolone resistance, qnr, Vibrio cholerae, superintegron repeats, class 1 integron, Amazon region, Brazil, dispatch

## Abstract

A novel *qnr* determinant emerged in ciprofloxacin-resistant *Vibrio cholerae* O1 from the Amazon region of Brazil. This *qnr*VC1 was in a typical class 1 integron. Its *attC* showed 89% identity with *V. parahaemolyticus* superintegron repeats. Analysis showed *V. cholerae* O1 carrying *qnr*VC2 associated with a *V*. *cholerae* superintegron repeat.

Quinolones are antimicrobial drugs effective against gram-negative bacteria. These drugs have been widely used as alternatives to conventional drug therapy for treating *Mycobacterium* spp. and *Neisseria* spp. infections. Quinolone resistance is frequently caused by mutations in DNA gyrase and topoisomerase IV chromosomal genes and an increased activity of efflux pumps that reduce intracellular concentrations of the drug (*1*).

Resistance to these drugs has been attributed to plasmid-mediated *qnr* genes. These genes are found mainly in *Enterobacteriaceae* and affect the dynamics of development and acquisition of quinolone resistance. Until now, all plasmid-borne *qnr* determinants were associated with atypical *sul*-type integrons, which are characterized by a duplication of a 3′ conserved segment, and a putative recombinase known as open reading frame (ORF) 513 (*2*). In contrast to typical gene cassettes, these *qnr* genes lack the *attC* site and, consequently, are unable to move by class 1 integrase excision. Qnr-like proteins coded by chromosomal genes have been found in *Vibrionaceae*, and this family has been identified as the source of *qnr* genes (*3*,*4*). However, no *qnr* homolog was found in any *Vibrio cholerae* genome sequenced.

Members of the family *Vibrionaceae* are characterized by superintegrons (SIs), which are chromosomal genetic elements containing a variety of gene cassettes with nearly identical *attC* sites (e.g., *V*. *cholerae* SI repeats) (*5*). We report 2 new *qnr*-like genes (*qnr*VC1 and *qnr*VC2) in typical class 1 integrons from clinical strains of *V*. *cholerae* O1.

## The Study

*V*. *cholerae* O1 isolates from the cholera epidemic in Brazil (1991–2000) were screened for antimicrobial drug resistance and class 1 integrons. VC627, a clinical strain from the Amazon region isolated in 1998, was resistant to ciprofloxacin, in contrast to other isolates. This strain was the only one positive for class 1 integrase but not for *qac*EΔ1 and *sul*I genes (*6*). Sequence analysis of the variable region of this class 1 integron showed an *aad*A2 gene cassette and an ORF similar to *qnr* determinants. Its deduced amino acid sequence showed 69% sequence identity with a chromosomal protein of *Photobacterium profundum*, which was recently identified as a quinolone-resistance determinant (QnrPP) (*3*) and 57% identity with QnrA1, a plasmid-encoded protein found in *Enterobacteriaceae* (*2*). This new *qnr*-like gene in *V*. *cholerae* was named *qnr*VC1 (EU436855).

Qnr proteins belong to the pentapeptide repeat family, which consists of uninterrupted pentapeptide repeats comprising 92% of the sequence and play a role in DNA gyrase protection (*7*). These repeat features were observed in the QnrVC1 deduced amino acid sequence with the consensus sequence [AC][DN][LF]XX (amino acids in brackets are found more frequently and XX indicates other amino acids) (*8*) (Appendix Figure), with a large proportion of serine residues in the fourth position (first X), as observed by Robicsek et al. (*2*).

The *qnr*VC homologs in chromosomes of other bacterial species were investigated by using BLAST analysis (www.ncbi.nlm.nih.gov/blast/Blast.cgi). Similar to other reports of such homologs (*3*,*4*), we found Qnr-like proteins in recently published genomes from different families, including other members of *Vibrionaceae* (Figure). Analysis in conjunction with informatics capabilities (in silico analysis) showed a 657-nt sequence in a class 1 integron of *V*. *cholerae* O1 isolated in Vietnam in 2004 (AB200915). This ORF had 75% nt similarity with *qnr*VC1, and it was designated *qnr*VC2.

**Figure:**
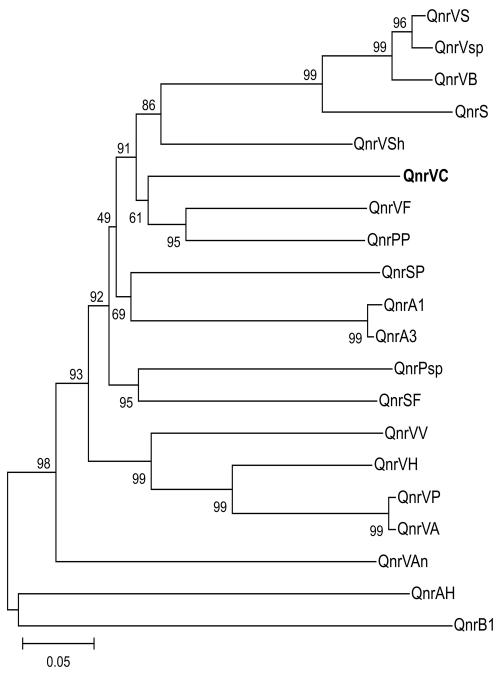
Genetic relationships of plasmid- and chromosome-encoded *qnr* proteins. Species and GenBank accession nos. are as follows. QnrVS (*Vibrio splendidus*, EAP95542), QnrVsp (*Vibrio* sp., EAQ55748), QnrS1 (*Shigella flexneri*, BAD88776), QnrVC (*V*. *cholerae*, strain 627; EU436855; this work, shown in **boldface**); QnrPP (*Photobacterium profundum*, YP132629), QnrVF (*V*. *fisheri*, AAW85819), QnrSP (*Shewanella pealeana*, EAV99957), QnrA1 (*Escherichia coli*, AAY46800), QnrA3 (*S*. *algae*, AAZ04782), QnrPsp (*Psychromonas* sp., EAS39797), QnrSF (*S*. *frigidimarina*, ABI71948), QnrVV (*V*. *vulnificus*, AAO07889), QnrVP (*V*. *parahaemolyticus*, BAC61438), QnrVA (*V*. *alginolyticus*, EAS75285), QnrVAn (*V*. *angustum*, EAS64891), QnrAH (*Aeromonas hydrophila*, ABK38882), QnrB1 (*Klebsiella pneumoniae*, ABG82188), QnrVSh (*V*. *shilonii*, EDL55273), QnrVB (*Vibrionales bacterium*, EDK31146), QnrVH (*V*. *harveyi*, EDL69958). Support of the branching order was determined by 1,000 interior branch test replicates. The distance-based tree was generated by using p distance with the neighbor-joining method with MEGA version 3.1 (www.megasoftware.net). Values along the horizontal lines are the interior-branch test percentages after testing 1,000 topologies. Scale bar indicates the number of substitutions per alignment site, which is reflected by branch lengths.

The *qnr*VC gene cassettes are characterized by *attC* sites, which are absent in all others *qnr* genes already identified. Supporting the hypothesis of *Vibrionaceae* as the source of *qnr* genes (*3*), the *qnr*VC1 *attC* site has 89% identity with *V*. *parahaemolyticus* repeats, and the *qnr*VC2 putative *attC* site has 96% identity with a *V*. *cholerae* repeat sequence, both of which are characteristic structures of SIs. Rowe-Magnus et al. (*5*) reported that SI repeat recombination sites are species specific and these elements would be the source of gene cassettes found in drug-resistance integrons, such as class 1 integrons. Therefore, we hypothesize that *qnr*VC1 gene cassette could have originated from a *V*. *parahaemolyticus* SI, whereas *qnr*VC2 originated in *V*. *cholerae*. Curiously, no chromosomal *qnr* genes identified in *Vibrionaceae* were associated with SIs.

The *qnr*VC1 gene showed high amino acid divergence compared with all *qnr* genes described, with similarities ranging from 44% to 69%. The *qnr*VC1 had a G + C content of 36.8%, which is considerably different from that of the *V*. *cholerae* genome (47.5%) and is evidence of horizontal gene transfer.

Promoter Pc, which is found in the 5′ conserved segment of the integron carrying *qnr*VC1, was identical to the hybrid configuration described (*9*,*10*) and is defined by 1 point mutation in the canonical –35 region. The hybrid Pc version has 20-fold lower promoter activity than that of the wild-type (strong) promoter. However, another study verified expression of genes inserted into integrons under the control of a hybrid Pc configuration (*11*). Moreover, in silico analysis detected a putative promoter (–35 TTGAGA [17 nt] –10 TAGTCT) in the 5′ untranslated region of the *qnr*VC1 gene cassette. Therefore, our findings characterize conditions for *qnr*VC1 expression in the class 1 integron.

Quinolones have excellent antimicrobial activity against *V*. *cholerae* (*12*). Strain VC627 shows a 10-fold increase in resistance to ciprofloxacin (MIC 0.25 μg/mL) compared with strains from the seventh pandemic lineage in Brazil (MIC 0.02 μg/mL). This increased resistance was also observed in a *Shigella* *flexneri* strain harboring *qnr*S (*13*,*14*) and *V*. *splendidus* harboring chromosomal *qnr*VS1 and *qnr*VS2 (*4*). This MIC value resembles that caused by *gyr*A mutations in *V*. *cholerae* ciprofloxacin-resistant strains (*15*). Therefore, dissemination of *qnr* genes by lateral gene transfer may determine, in a 1-step fashion, the same drug-resistance profile caused by acquired mutations in housekeeping genes.

## Conclusions

Our study reports characterization of 2 new *qnr* genes associated with SI *attC* sites in typical class 1 integrons and their emergence in *V*. *cholerae*. These genetic features have not been observed in either chromosomal- or plasmid-borne *qnr* determinants already described.

Our study suggests that, at least in *Vibrionaceae*, quinolone-resistance genes were mobilized from SIs by class 1 integrase activity because *qnr*VC1 and *qnr*VC2 *attC* elements are similar to *V*. *parahaemolyticus* and *V*. *cholerae* SI repeats. These findings corroborate the hypothesis that SIs are a source of cassettes present in drug-resistance integrons (*5*). These elements are part of a reasonable scenario for a 1-step acquisition of quinolone resistance by bacteria (e.g., *Mycobacterium* spp. and *Neisseria* spp.). This acquisition may jeopardize treatment and control and have adverse consequences on infections caused by these organisms.

## Supplementary Material

Appendix Figure Deduced amino acid sequence comparison of QnrVC1 from class 1 integron with plasmid- and chromosomal-mediated Qnr determinants. The glycine residue linking the 2 domains of the pentapeptide proteins is indicated by an arrow. Identical residues are highlighted and amino acid substitutions are in boldface. Species and GenBank accession nos. are as follows: QnrVS (Vibrio splendidus, EAP95542), QnrVsp (Vibrio sp., EAQ55748), QnrS1 (Shigella flexneri, BAD88776), QnrVC (V. cholerae strain 627, EU436855; this work); QnrPP (Photobacterium profundum, YP132629), QnrVF (V. fisheri, AAW85819), QnrSP (Shewanella pealeana, EAV99957), QnrA1 (Escherichia coli, AAY46800), QnrA3 (S. algae, AAZ04782), QnrPsp (Psychromonas sp., EAS39797), QnrSF (S. frigidimarina, ABI71948), QnrVV (V. vulnificus, AAO07889), QnrVP (V. parahaemolyticus, BAC61438), QnrVA (V. alginolyticus, EAS75285), QnrVAn (V. angustum, EAS64891), QnrAH (Aeromonas hydrophila, ABK38882), QnrB1 (Klebsiella pneumoniae, ABG82188), QnrVSh (V. shilonii, EDL55273), QnrVB (Vibrionales bacterium, EDK31146), QnrVH (V. harveyi, EDL69958).
