# Supplementary figures and images for: New qnr Gene Cassettes Associated with Superintegron Repeats in Vibrio cholerae O1
Source: Emerg Infect Dis. 2008 Jul;14(7):1129–31. doi: 10.3201/eid1407.080132 (PMC2600354; doi:10.3201/eid1407.080132)

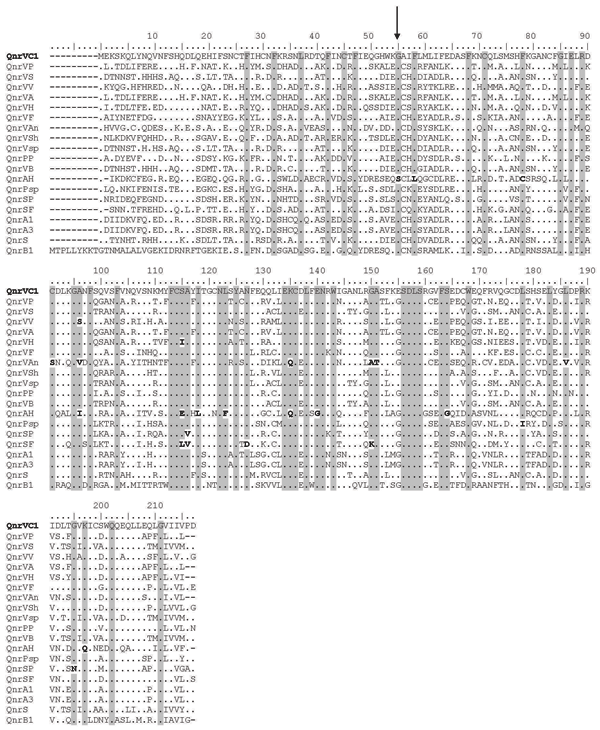

Supplement: Appendix Figure — Deduced amino acid sequence comparison of QnrVC1 from class 1 integron with plasmid- and chromosomal-mediated Qnr determinants. The glycine residue linking the 2 domains of the pentapeptide proteins is indicated by an arrow. Identical residues are highlighted and amino acid substitutions are in boldface. Species and GenBank accession nos. are as follows: QnrVS (Vibrio splendidus, EAP95542), QnrVsp (Vibrio sp., EAQ55748), QnrS1 (Shigella flexneri, BAD88776), QnrVC (V. cholerae strain 627, EU436855; this work); QnrPP (Photobacterium profundum, YP132629), QnrVF (V. fisheri, AAW85819), QnrSP (Shewanella pealeana, EAV99957), QnrA1 (Escherichia coli, AAY46800), QnrA3 (S. algae, AAZ04782), QnrPsp (Psychromonas sp., EAS39797), QnrSF (S. frigidimarina, ABI71948), QnrVV (V. vulnificus, AAO07889), QnrVP (V. parahaemolyticus, BAC61438), QnrVA (V. alginolyticus, EAS75285), QnrVAn (V. angustum, EAS64891), QnrAH (Aeromonas hydrophila, ABK38882), QnrB1 (Klebsiella pneumoniae, ABG82188), QnrVSh (V. shilonii, EDL55273), QnrVB (Vibrionales bacterium, EDK31146), QnrVH (V. harveyi, EDL69958). [file 08-0132_app-s1.gif]
